# Primary gastric Hodgkin's lymphoma

**DOI:** 10.1186/1477-7819-5-119

**Published:** 2007-10-21

**Authors:** Fahad S Hossain, Yashwant Koak, Farrukh H Khan

**Affiliations:** 1 Department of Surgery, Queen Mary's Sidcup Hospital, Frognal Avenue, Sidcup, Kent, DA14 6LT, UK; 2 Department of Surgery, Basildon University Hospital, Nethermayne, Basildon, Essex, SS19 5NL, UK

## Abstract

**Background:**

Primary Hodgkin's disease of the stomach is an extremely rare entity. Nearly all cases of primary gastric lymphoma are of the non-Hodgkin's variety. Diagnoses in such cases are difficult due to considerable histological similarities between the 2 disease entities.

**Case presentation:**

We report the case of a 77 year old lady with a 1 year history of weight loss and poor appetite. Physical examination was unremarkable. Subsequent multiple upper GI endoscopies revealed a large malignant looking ulcer which was deemed to be histologically benign. Following CT imaging the patient underwent a radical gastrectomy. Postoperatively histology and immunohistochemistry failed to confirm a diagnosis. As such a second opinion was sought. Employing an extended array of immunohistological staining a diagnosis of 'Classical Hodgkin's' disease of the stomach was achieved.

**Conclusion:**

Our case illustrates the significant difficulties in achieving a rare diagnosis of primary Hodgkin's lymphoma of the stomach. The non-specific nature of symptoms and a lack of histological features make a preoperative diagnosis extremely difficult. While immunohistochemistry is widely employed in aiding the evaluation of such cases, one should be wary of the considerable overlap in differentiating between Hodgkin's and non-Hodgkin's disease entities using this technique.

## Background

The incidence of primary Hodgkin's disease of the stomach is rare. The data from National Cancer Institute between 1953 and 1990 identified only 6 cases of histologically reconfirmed Hodgkin's disease of gastrointestinal tract [1].

The diagnosis of Hodgkin's disease depends primarily on the detection of Reed-Sternberg cells by light microscopy, but Reed Sternberg like cells are also noted in peripheral T cell lymphoma, CD30 positive large cell lymphoma and malignant histiocytosis [2-4]. Hence immunohistochemical staining in addition to conventional histological studies have recently been employed for differential diagnosis of these similar diseases [5]. This is important as accurate diagnosis of Hodgkin's disease and non-Hodgkin's lymphoma is essential in choosing the most appropriate therapies. This paper reports a case of primary Hodgkin's disease of the stomach after definitive postoperative diagnosis following histopathological and immunohistochemical study.

## Case presentation

A 77 year old Caucasian female was admitted to Basildon University Hospital with a one year history of poor appetite and weight loss of 2 stones accounting for a quarter of her body weight. She did not complain of any abdominal pain, nausea, vomiting, haematemesis or malaena. Apart from a history of clinical depression her past medical, family and social history was unremarkable.

Physical examination revealed a well looking elderly lady with normal vital signs. There was no peripheral lymphadenopathy and examination of the abdomen was unremarkable. The liver and spleen were not palpable and there was no ascites. Laboratory investigations showed FBC haematocrit of 39%, WBC of 4.9 × 10^9^/L and Hb of 13.9 g/dL. Liver enzymes and other relevant biochemistry tests were normal.

Subsequent multiple upper GI endoscopies revealed a large malignant ulcer of the greater curve of the stomach with multiple satellite lesions extending proximally. Histological examination of biopsies taken showed the ulcer to be benign. A CT scan of the chest, abdomen and pelvis demonstrated a tumour extending from the cardia into the body of the stomach with a large polypoid component. There was no evidence of lymphadenopathy in the vicinity of the tumour or in the para-aortic region. The liver, spleen and the pancreas were normal. Furthermore there was no evidence of mediastinal or hilar lymphadenopathy or pulmonary deposits.

Staging laparoscopy did not reveal any intra abdominal seedlings, ascites or lymphadenopathy. At laparotomy intra-operative findings were of a large tumour involving the lesser curvature, the body and the greater curvature of the stomach with a 7 × 6 × 1 cm ulcer on the greater curvature infiltrating into the stomach wall. A radical gastrectomy was performed with *en-bloc *resection of 4 lymph nodes from the lesser curvature, 5 from the greater curvature and one from the greater omentum.

**Figure 1:**
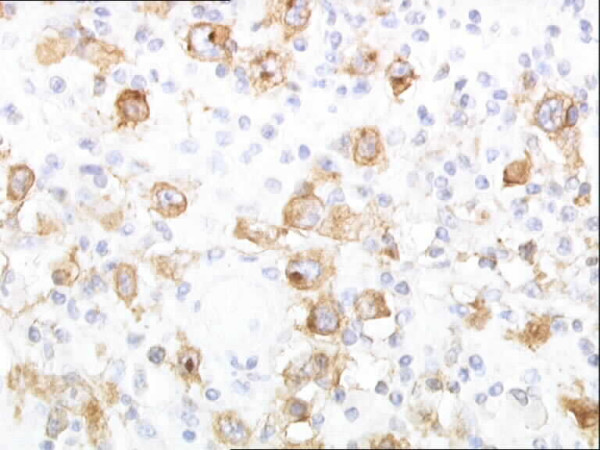
Photomicrograph with immunohistochemical staining showing CD30 positive large cells in inflamed gastric mucosa.

**Figure 2:**
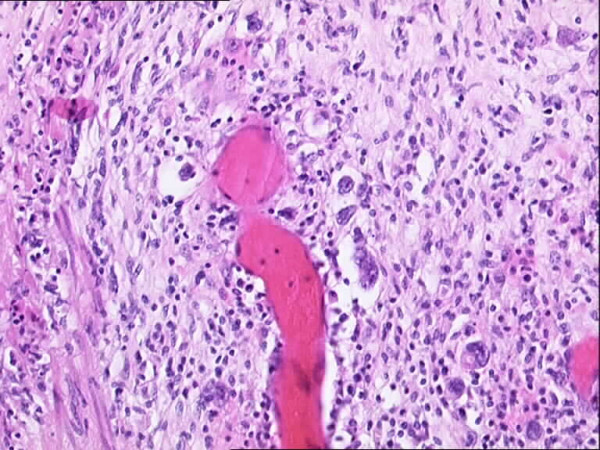
Photomicrograph showing mononuclear and bi-lobed variants of Reed-Sternberg cells as in 'Classical Hodgkins's' disease on a background of ulcerated gastric mucosa.

Initial histopathological examination of the resected stomach from the ulcer site at the local hospital showed abundant lymphocytic infiltration composed of lymphocytes and eosinophils. Immunohistochemical staining showed numerous CD30 positive large cells (Figure 1). A preliminary diagnosis of anaplastic large cell lymphoma was considered. Specimens were sent for a second opinion to University College London Hospital. A polymorphous lymphoid infiltrate was identified including scattered large cells showing Reed-Sternberg morphology (Figure 2). A panel of immunohistochemical markers were stained for. A predominant expression of CD30, CD15 and EBV-LMP1 were seen in the cells while being weakly positive for CD20. Staining for CD79a, CD3, bcl-2 and bcl-6 were negative. A diagnosis of "Classical Hodgkin's disease of the stomach" was made.

The postoperative period was uneventful and follow-up with repeat CT scans at 3, 6 and 12 months revealed no evidence of residual disease or relapse. No chemotherapy was initiated; however a plan to administer a regime of chlorambucil and prednisolone was made should there be a relapse of disease on further follow-up.

## Discussion

Lymphoma of the gastrointestinal tract is seen more commonly in the context of disseminated disease. Primary gastric Hodgkin's disease is extremely rare. In a review by Colluci et al. of 721 patients with primary gastric lymphoma between 1973 and 1990, only 17 were diagnosed as the Hodgkin's variety [6]. Further to this a literature review of the Medline and Embase databases reveal only a further 6 cases of Primary Hodgkin's disease of the stomach between 1990 and August 2007 excluding ours (Table 1). With the uncertainty of histological diagnosis and hence the development of immunohistochemical techniques for gastrointestinal Hodgkin's disease, it has been reported that the frequency of primary Hodgkin's disease of the stomach is probably even less than 1% of all gastric lymphomas [7]. This is supported in various studies where originally diagnosed cases of Hodgkin's disease were all reclassified as non-Hodgkin's lymphoma of a large type after re-examination [8]. As such non-Hodgkin's lymphoma is considered the predominant gastric lymphoid malignancy [9], which has increased in incidence in contrast to gastric carcinoma, which has declined in incidence over the past few decades [10].

**Table 1:** Reported cases of primary gastric Hodgkin's Lymphoma showing immuno/histological characteristics and pre-operative diagnosis between 1990 and August 2007.

**Author**	**Year**	**Immuno/Histology Immunohistochemistry Histology**		**Pre op endoscopic biopsy**	**Cases**	**Ref.**
Ogawa et al	1995	CD30^+^, CD45^+ ^CD15^-^, EMA^-^	RS cells	B-cell malignant lymphoma	1	5
Mori et al.	1995	CD30^+^, CD3^-^, CD15 not done	Atypical multinucleate cells	Ulcerative lesion	1	18
Zippel et al.	1997	-	RS cells	Adenocarcinoma	1	19
Venizelos et al.	2005	CD30^+^, CD15^+^, EMA^- ^CD20^+^(weak), CD79a^-^	Atypical mononuclear cells & RS cells	Chronic Gastritis	1	20
Penázová et al	2007	CD15^+^, CD30^+^	Large mononuclear cells & RS cells	Hodgkin's Disease	1	21
Saito et al	2007	CD30^+^, CD20^+^, CD79a^+^, CD3^-^, CD15^-^, EMA^-^, Oct-2^+^, Bob-1^+^	Atypical multinuclear cells & RS cells	Inconclusive biopsy	1	22

The stomach is the most common site of primary extranodal lymphoma in adults [10]. In a previous study Dawson *et al*., [11] proposed a set of criteria for the diagnosis of primary gastrointestinal lymphoma including a) absence of peripheral lymphadenopathy at the time of presentation, b) lack of enlarged mediastinal lymph nodes, c) a normal total WBC and differential, d) predominance of the bowel lesion at the time of laparotomy with the only lymph nodes obviously affected being those in its immediate vicinity, e) the liver and spleen not showing any lymphomatous involvement. Our current case fulfils all these criteria.

Difficulties in pre-operative diagnosis of Hodgkin's disease of the stomach have been cited before (Table 1). This is clearly illustrated in our case. Diagnostic endoscopy usually reveals non-specific gastritis or peptic ulcers with mass lesions being unusual [12] as was the case in our patient. Certainly a misdiagnosis of large cell lymphoma was made even after surgery in our patient. This can be attributed to the relatively low rate of Reed-Sternberg cells in biopsy specimens and the increasing prevalence of histologically similar diseases. While it is clear that a histological diagnosis may be inadequate in cases of gastric Hodgkin's disease due to the presence of many disease entities exhibiting Reed-Sternberg like morphology [2-4] one must note that considerable uncertainty may also be encountered in immunohistochemical investigation. In the case of our patient the specimens initially immunologically stained were positive for CD30 and hence a misdiagnosis of CD30 positive large cell lymphoma was made. Apart from large cell lymphomas the CD30 antigen is expressed in various cell lineages such as malignant histiocytosis, plasmacytoma, some non-Hodgkin lymphomas other than large cell lymphoma, lymphomatoid papulosis and certainly Hodgkin's disease [3,13]. A full panel of immunohistochemical markers is hence essential to make an accurate diagnosis of gastric Hodgkin's disease. Our patient showed co expression of both CD30 and CD15, which is commonly associated with Hodgkin's disease [8]. Variable expression of CD20 and even less commonly CD79a in cases of Hodgkin's disease has also been demonstrated [14]. In our case post surgery specimens stained weakly for CD20 and were entirely negative for CD79a.

The epidemiological and pathogenic association of classic Hodgkin's disease with the Epstein-Barr virus has been well established [15,16]. LMP1 (Latent Membrane Protein 1) an essential EBV protein commonly expressed and associated with the pathogenesis of Hodgkin's disease [17] was also positively stained for in our case. While its presence is not diagnostic of primary Hodgkin's disease of the stomach it does confer some indication to its aetiology.

Hodgkin's disease of the stomach has been treated by surgery while post operative chemotherapy has been employed for systemic disease. Post operative therapy may be necessary because gastric Hodgkin's disease may represent one expression of systemic lymphoma and another portion of the lymphoid system may develop malignancy post operatively [5]. Our patient underwent a radical gastrectomy but on follow-up no recurrent disease was seen. Due to the nature of the disease, relapse is more than likely and hence a plan for further follow-up and chemotherapy was made.

## Conclusion

Prognosis of Hodgkin's disease of the stomach is poor with 45–60% of patients dying within the first year of diagnosis [5]. This has been attributed to the difficulties in differential diagnosis where gastric Hodgkin's disease is misdiagnosed.

## Competing interests

The author(s) declare that they have no competing interests.

## Authors' contributions

FSH conducted a literature search and drafted the manuscript

YK assisted during the surgery and editing the manuscript

FHK consultant surgeon who carried out the surgical procedure and made necessary corrections and proof read the final manuscript.

All authors read and approved the final manuscript.
